# Off-label prescribing of quetiapine in HMP Elmley, a Category B remand prison: a re-audit

**DOI:** 10.1192/bjo.2021.262

**Published:** 2021-06-18

**Authors:** Nosa Igbinomwanhia, Kathleen McCurdy

**Affiliations:** Oxleas NHS Foundation Trust

## Abstract

**Aims:**

This was a re-audit of off-label prescribing of quetiapine in order to identify the number of patients on off-label quetiapine in HMP Elmley, to monitor compliance by the Mental Health Inreach Team (MHIRT) psychiatrists with the Royal College of Psychiatrists guideline on off-license prescribing, to compare findings with the baseline audit and to identify areas for improvement.

**Method:**

All patients on quetiapine in HMP Elmley were identified and their electronic patient record was reviewed against the standards outlined in the Royal College of Psychiatrists “Use of licensed medicines for unlicensed applications in psychiatric practice (2nd edition).

**Result:**

There were 60 residents on off-license quetiapine prescription in HMP Elmley.

Of this number, four had their prescription initiated by a general practitioner, either while in prison or in the community. Two residents were on quetiapine first prescribed while they were on admission in hospital. 5 patients had been initiated by the MHIRT psychiatrists. 38 residents were commenced off-license quetiapine by another psychiatrist, either while they were in the community or in another prison. In 17 patients, electronic records were inadequate to determine who had prescribed the quetiapine.

The number of inmates prescribed off-label quetiapine in HMP Elmley had dropped from 82 to 60 in the 1 year since the initial audit. Of these figures, prescriptions initiated by the MHIRT psychiatrists, had dropped from 28.1% (23/82) to 8.3% (5/60).

For those prescribed quetiapine by the HMP Elmley psychiatrists, notes were audited against the RCPsych guidelines:

- Licensed medication was considered first in 80.0%
- Risks and benefits were considered and documented in 80.0%
- The benefits and potential risks were explained to patient in 80.0%
- There was documentation of informed consent in 80.0%
- Quetiapine was started at a low dose and monitored in 100%
- No residents required withdrawal of medication due to ineffectiveness or adverse effects.
- Baseline physical health assessment was performed in 80.0%, though all had an ECG done.

**Conclusion:**

Over the past year there has been an improvement in off-label antipsychotic prescribing practice within the MHIRT.

However, the number of off-label antipsychotic prescriptions still remains high throughout the prison. There should be continued effort at minimizing off-label prescribing within the MHIRT, monitored by auditing. However, work needs to be done jointly with other prescribers, such as GP colleagues, in order to avoid unnecessary prescriptions and to monitor regularly the physical and mental health of those on off-label quetiapine.

